# Metastatic Pulmonary Calcinosis and Leukocytoclastic Vasculitis in a Patient with Multiple Myeloma

**DOI:** 10.5505/tjh.2012.23600

**Published:** 2012-12-05

**Authors:** Seçkin Çağırgan, Nur Soyer, Filiz Vural, Güray Saydam, Ilgın Yıldırım Şimşir, Ayhan Dönmez, Taner Akalın, Selen Biçeroğlu, Murat Tombuloğlu

**Affiliations:** 1 Ege University, School of Medicine, Department of Hematology, İzmir, Turkey; 2 Ege University, School of Medicine, Department of Pathology, İzmir, Turkey; 3 Ege University, School of Medicine, Department of Radiology, İzmir, Turkey

**Keywords:** Multiple myeloma, Metastatic calcification, Leukocytoclastic vasculitis

## Abstract

Both leukocytoclastic vasculitis and metastatic pulmonary calcification are conditions that rarely occur during the course of multiple myeloma. We present a multiple myeloma patient that had severe dyspnea due to metastatic pulmonary calcinosis, and ulceronecrotic skin lesions caused by leukocytoclastic vasculitis. After 3 courses of standarddose chemotherapy all skin and pulmonary lesions disappeared. Autologous peripheral stem cell transplantation was performed and during 1 year of follow-up the patient was in complete remission; after 1 year, laboratory test results indicated disease relapse. Although the patient was treated with bortezomib and dexamethasone, the disease progressed. Non-myeloablative allogeneic stem cell transplantation was performed, but despite of all treatment the patient died due to disease progression.

**Conflict of interest:**None declared.

## Abstract (tr)

Hem lökositoklastik vaskülit hemde metastatik pulmoner kalsifikasyon, multipl myelomda nadir olarak görülmektedir. Biz, lökositoklastik vaskulit nedeniyle gelişen ülseronekrotik cilt lezyonları ve ciddi dispneye yol açan metastatik pulmoner kalsifikasyonla tanısı konan bir multipl myelom hastasını sunmak istedik. Üç kür standart doz kemoterapi sonrası hastanın hem cilt lezyonları hemde pulmoner lezyonları düzeldi. Otolog kök hücre nakli yapılan hasta 1 yıl tam remisyonda izlendi. Daha sonra laboratuvar testlerinde relaps saptandı. Hastaya bortezomib ve deksametazon başlandı. Bu tedaviye rağmen progresyonu olan hastaya myeloablatif olmayan rejimle allojeneik kök hücre nakli yapıldı. Bütün tedavilere rağmen hasta progresif hastalıkla kaybedildi.

## INTRODUCTION

Multiple myeloma is a clonal malignant plasma cell disease. Manifestations of the disease develop due to bone marrow and skeletal infiltration, and immunoglobulin production by malignant plasma cells [1]. Leukocytoclastic vasculitis is a systemic vasculitis characterized by involvement of small vessels in the skin that present as palpable purpura. It may present with systemic manifestations, such as fever, arthralgia as a finding of arthritis, and less commonly renal, neurological, and gastrointestinal functional compromise [2,3,4]. This disorder is related to hypersensitivity mechanisms caused by various antigens, primarily infections, drugs, or autoantigens in connective tissue diseases and malignant neoplasms [3]. Most reports of cutaneous leukocytoclastic vasculitis occurred in patients with hematological malignancies and presented as paraneoplastic vasculitis [3,4,5,6]. The incidence of paraneoplastic vasculitis in patients with multiple myeloma is approximately 0.8% [3]. 

Metastatic pulmonary calcification is the deposition of calcium in pulmonary interstitium and parenchyma. Predisposing factors include chronic renal failure, hypercalcemia, and elevated tissue alkalinity [7,8]. Hypercalcemia is a common manifestation of multiple myeloma and, as such, metastatic calcification is an expected consequence; however, to the best of our knowledge an association between metastatic calcification and cutaneous leukocytoclastic vasculitis in patients with multiple myeloma has not been reported. Herein we present a multiple myeloma patient that presented with severe dyspnea due to metastatic pulmonary calcinosis, and ulceronecrotic skin lesions.

## CASE REPORT

Written informed consent was obtained from the patient. 

**Figure 1:**
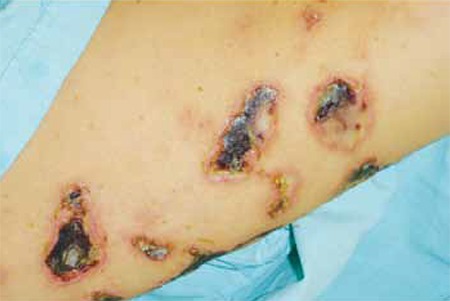
Well-delimited necrotic skin ulcers.

A 35-year-old female presented with severe dyspnea, back pain, and multiple painful purpuric ulceronecrotic skin lesions on her face and extremities in November 2005. She was hospitalized with the presumed diagnosis of respiratory failure based on findings of hypoxemia and scattered density changes on chest X-ray. Physical examination showed widespread fine rales in chest auscultation and diffuse palpable purpura of variously sized patches, some of which were necrotic, that formed well-delimited ulcers (Figure 1). The patient was afebrile and there were no signs of an infectious focus. 

Initial laboratory analysis results were as follows: hemoglobin: 7 g dL^–1^; hematocrit: 20.3%; leukocyte count: 12.1 x 10^9^ L ^–1^; platelet count: 123 x 10^9^ L ^–1^; serum urea 76 mg dL^–1^ (normal range: 10-50 mg dL^–1^); creatinine: 1.66 mg dL^–1^ (normal range: 0.6-1.1 mg dL^–1^); creatinine clearance: 26 mL s^–1^ (normal range: 70-145 mL s^–1^); calcium: 13.8 mg dL^–1^ (normal range: 8.2-10.4 mg dL^–1^); globulin: 5 g dL^–1^ (normal range: 2.5-3.5 g dL^–1^). Serum protein electrophoresis showed a monoclonal peak in the gammaglobulin zone. Serum immunoelectrophoretic analysis showed there was immunoglobulin G (IgG) and kappa chain fixation. Serum IgG, IgA, and IgM levels were 3349 mg dL^–1^ (normal range: 650-1600 mg dL^–1^), <25 mg dL^–1^ (normal range: 40-350 mg dL^–1^), and 27 mg dL^–1^ (normal range: 50-300 mg dL^–1^), respectively. Urinary kappa and lambda levels were 847 mg dL^–1^ (normal range: 138- 375 mg dL^–1^) and 16 mg dL^–1^ (normal range: 92-242 mg dL^–1^), respectively. Daily urinary protein excretion was 1 g d^–1^ and Bence-Jones proteinuria was negative. Analysis of a bone marrow biopsy specimen showed that there was 90% immature plasma cell infiltration. Multiple osteolytic lesions were observed in the skull, scapula, clavicle, humerus, femurs, and tibias. 

**Figure 2:**
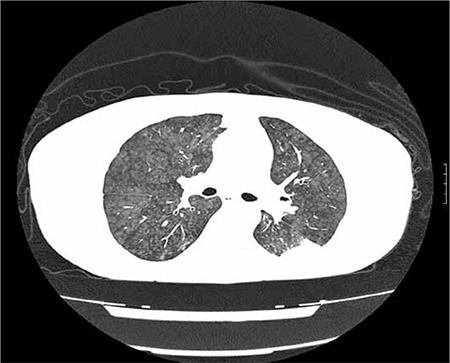
HRCT at the level of the upper lobes shows nodular ground glass opacities with a predominately centrilobular distribution.

**Figure 3:**
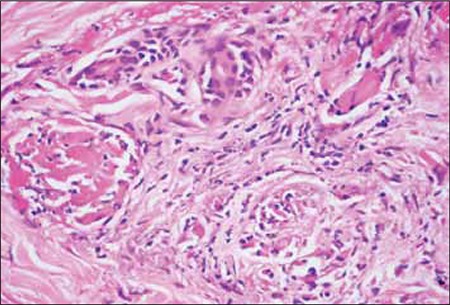
Leukocytoclastic vasculitis with widespread fibrin thromboses in small vessels (H&E, 200x).

The patient was diagnosed as stage III-A IgG-kappa multiple myeloma, according to Durie-Salmon classification. Thoracic high-resolution computed tomography (HRCT) showed bilateral widespread centrilobular acinar opacities and a ground-glass appearance, which was consistent with metastatic calcinosis of the lungs (Figure 2). Whole-body scintigraphy also showed metastatic calcium depositions in soft tissues. Biopsy specimens obtained from the skin lesions showed findings of leukocytoclastic vasculitis, as well as diffuse fibrin thromboses in small vessels and necrosis on the epidermis. Immunofluorescence examination showed the presence of fibrinogen in superficial dermis and vessel walls, as well as granular C3 deposition on the vessel walls (Figure 3). Calcium and amyloidal deposition was not detected based on histological examination with special Congo red and von Kossa dyes. 

The patient was treated with standard-dose VAD (vincristine, adriamycin, and dexamethasone) chemotherapy and zoledronic acid. All skin and pulmonary lesions disappeared after the third course of chemotherapy, and serum calcium and creatinine levels returned to normal. The patient achieved partial remission. Following the fourth course of chemotherapy, the patient was treated with high-dose melphalan (200 mg m–2) and autologous peripheral stem cell transplantation. During 1 year of post transplantation follow-up the patient was in complete remission. One year after the transplantation laboratory test results showed relapse of multiple myeloma and that the patient’s IgG level was 3344 mg dL^–1^. She was treated with 3 courses of bortezomib and dexamethasone, after which time her serum IgG level increased to 4199 mg dL^–1^. Because of disease progression she underwent non-myeloablative allogeneic stem cell transplantation (busulphan 3.2 mg·kg·d^–1^ for 2 d, cyclophosphamide 350 mg m–2 for 3 d, and fludarabine 30 mg m–2 for 3 d) from an HLA fullymatched sibling donor. Following the transplantation acute graft versus host disease was not observed; however, disease response was not achieved. Three months after the transplantation donor lymphocyte infusion was administered in order to control the progression of disease, but despite all treatment the patient died at home due to disease progression in October 2007.

## DISCUSSION

The presented patient presented with severe dyspnea and ulceronecrotic skin lesions, and was diagnosed as multiple myeloma. A skin biopsy specimen obtained from ulceronecrotic lesions showed leukocytoclastic vasculitis and HRCT of the lungs showed metastatic pulmonary calcification. To the best of our knowledge this is the first case reported with these 3 findings. After 3 courses of standard first-line multiple myeloma chemotherapy, the patient achieved partial remission, and all skin and pulmonary lesions disappeared. 

Leukocytoclastic vasculitis is a paraneoplastic syndrome, which is observed most commonly in cases of hematological malignancy, such as lymphoid neoplasm or myelodysplastic syndrome [4,6]. Leukocytoclastic vasculitis rarely occurs during the course of multiple myeloma [2]. This vasculitis is an inflammatory necrotizing condition of the superficial dermal vessels, and is characterized by neutrophilic, angiocentric, segmental inflammation with endothelial cell injury and fibrinoid necrosis of the blood vessel walls [9]. Clinical symptoms are variable and include palpable purpura, and hemorrhagic-necrotizing, bullous, nodular, and urticarial lesions [10]. 

Sanchez et al. [2] reported a case of multiple myeloma associated with paraneoplastic vasculitis and reviewed 8 other reported patients. In all, 6 of the 9 patients were diagnosed as type IgA myeloma. All the patients had palpable purpura involving the legs and/or trunk, and 2 patients had ulceronecrotic lesions, as in the presented case. Skin lesions in 6 of the patients improved after treatment of multiple myeloma. Bayer-Garner et al. reported 8 patients with multiple myeloma that developed leukocytoclastic vasculitis [11]. They reported that 4 of the patients were diagnosed as IgG myeloma with diffuse skin lesions, as was the presented case. 

Metastatic pulmonary calcification generally occurs in patients with hypercalcemia [12]; multiple myeloma is less commonly a cause of metastatic pulmonary calcification. Generally, patients with metastatic pulmonary calcification are asymptomatic, but restrictive lung function, decreased diffusing capacity, hypoxemia, and respiratory failure may occur [12]. Plain radiography of the chest is usually normal. HRCT generally shows centrilobular ground glass nodular opacities. In cases of metastatic calcification calcium deposits may accumulate to such tissues as lung, stomach, skin, and kidney [13,14]. Weber et al. reported a multiple myeloma patient with metastatic pulmonary calcification. After treatment of multiple myeloma, pulmonary infiltration decreased, as in the presented case [15]. Marchiori et al. [12] reported 3 patients who presented with metastatic pulmonary calcification with unusual HRCT findings, 1 of which died due to diffuse metastatic calcification in the parenchyma of major organs, including the heart, lungs, kidneys, meninges, and skin, and was diagnosed as multiple myeloma during autopsy. Another study reported 4 cases diagnosed as paraneoplastic hypercalcemia with metastatic calcification, based on autopsy, 1 of which presented with paraneoplastic hypercalcemia and was diagnosed as multiple myeloma [16]. Most of the reported cases of metastatic calcinosis died early because of acute pulmonary failure or because the underlying disease was refractory to treatment; even though symptoms resolved following treatment, they died due to disease progression. 

In conclusion, both leukocytoclastic vasculitis and metastatic pulmonary calcification are conditions that rarely occur during the course of multiple myeloma. Treatment of multiple myeloma may improve skin lesions and metastatic calcification, but the prognosis appears to be poor despite administration of the most effective treatments. 

**Conflict of Interest Statement**

The authors of this paper have no conflicts of interest, including specific financial interests, relationships, and/ or affiliations relevant to the subject matter or materials included.
